# Children with low working memory and children with ADHD: same or different?

**DOI:** 10.3389/fnhum.2014.00976

**Published:** 2014-12-09

**Authors:** Joni Holmes, Kerry A. Hilton, Maurice Place, Tracy P. Alloway, Julian G. Elliott, Susan E. Gathercole

**Affiliations:** ^1^Medical Research Council Cognition and Brain Sciences UnitCambridge, UK; ^2^Tees, Esk and Wear Valleys NHS TrustDurham, UK; ^3^School of Health and Life Sciences, Northumbria UniversityNewcastle upon Tyne, UK; ^4^Department of Psychology, University of North FloridaJacksonville, FL, USA; ^5^School of Education, Durham UniversityDurham, UK

**Keywords:** ADHD, working memory, executive function, developmental disorders, intervention

## Abstract

The purpose of this study was to compare working memory (WM), executive function, academic ability, and problem classroom behaviors in children aged 8–11 years who were either identified via routine screening as having low WM, or had been diagnosed with ADHD. Standardized assessments of WM, executive function and reading and mathematics were administered to 83 children with ADHD, 50 children with low WM and 50 typically developing children. Teachers rated problem behaviors on checklists measuring attention, hyperactivity/impulsivity, oppositional behavior, and difficulties associated with executive function in the classroom. The ADHD and low WM groups had highly similar WM and executive function profiles, but were distinguished in two key respects: children with ADHD had higher levels of rated and observed impulsive behavior, and children with low WM had slower response times. Possible mechanisms for these common and distinct deficits are discussed.

## Introduction

Deficits in working memory (WM) are common in childhood. They are characteristic of children with specific learning difficulties in reading, mathematics, and language (e.g., Swanson and Ashbaker, 2000; Archibald and Gathercole, 2007; Szucs et al., 2013; Pimperton and Nation, 2014) and also of those with attention deficit hyperactivity disorder (ADHD) (e.g., Martinussen et al., 2005). Poor WM during development has begun to be investigated in its own right rather than as a secondary symptom of another disorder, and has been found to be closely associated both with low academic achievement (Gathercole and Alloway, 2008; Alloway et al., 2009) and with some of the attention problems typical of children with ADHD (Gathercole et al., 2008a). This study provides the first direct comparison of cognitive skills, executive functions, learning and behavior between children with low WM and those of the same age diagnosed with ADHD. The outcomes have direct implications both for the diagnosis and treatment of the broad range of cognitive and behavioral problems found in these two groups and for the interplay between inattention and poor WM in childhood.

WM is comprised of distinct but interacting cognitive and neural systems that coordinate higher-level attentional control and the temporary storage of information, providing vital ongoing support for complex cognitive activities (Baddeley, 2000; Unsworth and Engle, 2007; Cowan, 2010). There exist a variety of conceptualizations of the nature, structure, and function of WM (see Conway et al., 2007, for review). One important distinction between models is whether WM is conceived as a distinct multi-store workspace that includes an attentional component (e.g., Baddeley and Hitch, 1974; Baddeley, 2000) or is embedded within a broader limited-capacity system of controlled attention (e.g., Kane et al., 2001). In general, though, both frameworks provide adequate accounts of large bodies of empirical evidence (e.g., Cowan, 1995; Nee et al., 2008) and there is broad consensus that the storage-only capacities of short-term memory (STM) and the capacity-limited attentional control functions of WM can be distinguished (e.g., Shah and Miyake, 1999; Cowan, 2008).

Baddeley and Hitch's (1974; Baddeley, 2000) enduring multi-component model has provided a theoretical framework and set of methodologies that have been widely used for exploring WM in many cognitive developmental disorders including ADHD. In this, a domain-general limited-capacity central executive system provides executive control of attention. There are many parallels between this subsystem and the controlled attention view of WM (Kane et al., 2001). The central executive is part of a broader network of executive functions that includes inhibition, planning, and set switching and which relies on the same frontal brain networks to support flexible goal-directed behavior (Duncan and Owen, 2000; Miyake et al., 2001; St Clair-Thompson and Gathercole, 2006). Verbal and visuo-spatial short-term memory (STM) stores, and an integrative multi-modal episodic buffer, support the central executive (Baddeley, 2000). Assessments of these different components of WM are distinguished by whether or not they impose significant processing demands. Whereas STM tasks require the storage of information, tasks tapping the executive component (often termed complex span or WM tasks) involve significant processing in addition to storage (e.g., Daneman and Carpenter, 1980; Alloway et al., 2006). These components have been suggested to contribute to many everyday cognitive activities including following instructions (Yang et al., 2014), mental arithmetic (De Stefano and LeFevre, 2004) and the comprehension of language (Carretti et al., 2009).

Children selected on the basis of low scores on measures of WM that tax both the central executive and STM stores, such as backward digit span or listening span, typically perform relatively poorly on school-based evaluations of curriculum learning (e.g., Swanson and Sachse-Lee, 2001; Gathercole et al., 2003, 2004; Alloway and Alloway, 2010; Archibald et al., 2011). The majority have impairments in both reading and maths and in the classroom they frequently fail in activities that involve following instructions, storing information whilst engaged in other cognitively demanding activities, and place-keeping in complex tasks (Gathercole et al., 2006; Gathercole and Alloway, 2008). Laboratory tasks designed to simulate the high WM demands of classroom activities under more controlled conditions confirm these deficits (Engle et al., 1991; Gathercole et al., 2008b).

Children with poor WM are also reported by teachers to be inattentive and have short attention spans (Gathercole et al., 2008a; Alloway et al., 2009; Archibald et al., 2011). Similarly, adults with low WM report high levels mind-wandering under conditions of high cognitive load (Kane et al., 2007; McVay and Kane, 2009), and associations are found between poor WM and inattentive behavior in both typically developing children (Aronen et al., 2005; Lui and Tannock, 2007; Thorell, 2007) and those with poor comprehension skills (Pimperton and Nation, 2014). Children with low WM also exhibit problems in other areas of executive function. They are rated by teachers as being relatively poor in areas relating to WM, the ability to monitor work, the inhibition of impulsive responses, and in planning and organization (Gathercole et al., 2008a). On direct assessments of action planning and visual selective attention, they have also been reported to be impaired (St Clair-Thompson, 2011).

There is a high degree of overlap between this profile and the characteristics of children with combined-type ADHD, a disorder characterized both by abnormally high levels of both inattentive and hyperactive/impulsive behaviors (DSM 5 American Psychiatric Association, 2013). They too have impaired learning in reading and mathematics (Loe and Feldman, 2007), accompanied by WM difficulties (Martinussen et al., 2005; Willcutt et al., 2005a; Kofler et al., 2010) that have been linked with inattention (Willcutt et al., 2005a; Martinussen and Tannock, 2006).

The executive function problems in ADHD include response inhibition (e.g., Bledsoe et al., 2010), attentional switching (e.g., Oades and Christiansen, 2008), planning (e.g., Solanto et al., 2007), and sustained attention (e.g., Rubia et al., 2009). In the most common form of ADHD, the combined subtype, children also have excessively high levels of motor activity (hyperactivity) and impulsive behavior (Barkley, 1997; Halperin et al., 2008; Rapport et al., 2009). There is as yet no consensus regarding the origins of this complex profile of deficits. Some have argued that WM difficulties underlie other executive functions deficits in ADHD such as response inhibition (Rapport et al., 2008; Alderson et al., 2010). Others have suggested that executive function deficits (including WM) and problems of impulse control or aversion to reward delay represent impairments of two functionally distinct neurodevelopmental systems: “cool” cognitive—based executive functions that include inhibitory control and WM, and “hot” affective processes associated with aversion to delay that manifest as impulsive behavior (Tripp and Alsop, 2001; Castellanos et al., 2006).

Although the cognitive similarities between children with low WM and those with ADHD are striking, one marked difference is evident. Children with low WM do not exhibit excessive levels of motor activity and problems in impulse control that are core characteristics of ADHD (Gathercole et al., 2008a; Alloway et al., 2009). One possibility is that the two groups share a common deficit in the cool executive function system that is associated with attentional difficulties but that only those with ADHD have impairments in the hot executive system linked with hyperactivity and impulsivity. This hypothesis was investigated in the present study which, to our knowledge, is the first to compare directly the cognitive and behavioral characteristics of children who have poor WM but no ADHD diagnosis with those with ADHD. It was predicted that both groups would be impaired on direct measures of cool executive functions such as WM, planning and cognitive inhibitory control, and that they would be rated as being both inattentive and having elevated levels of other problem behaviors relating to these elements of high-level cognitive control. The ADHD group were expected to be differentiated by further problems in impulse control, and both hyperactive and impulsive behavior; the same difficulties were not predicted for the low WM group. Both direct assessments and teacher behavior ratings were obtained of a range of executive functions including WM and attentional control. Measures of IQ and learning were also included.

## Methods

### Participants

Three groups of children participated in the study. One group consisted of 83 children (71 boys) aged 8–11 years, with a clinical diagnosis of combined-type ADHD recruited through pediatric psychiatrists based in the North-East of England. All children had a clinical diagnosis of ADHD that included a psychosocial assessment, clinical and parent observer reports and a clinical assessment of the child's mental state. Inclusion criteria for the present study were i) a DSM 5 diagnosis of combined-type ADHD for 6 months or longer ii) aged between 8 and 11 years iii) no co-morbid Autistic Spectrum Disorders. The majority of the group was receiving fast-release stimulant medication for the condition: methylphenidate (*n* = 64), dexamphetamine (*n* = 2), dexedrine (*n* = 2), and imipramine (*n* = 1). Fifteen children were not taking medication.

Two further groups were recruited from a sample of 780 children aged 8–11 years attending 10 state primary schools in the same region who were screened on two tests of verbal WM: Listening Recall and Backward Digit Recall from the Automated Working Memory Assessment (AWMA; Alloway, 2007). Fifty children (30 boys) with standard scores below 86 on both tests were assigned to a low WM group, and a further 50 children (27 boys) with standard scores above 90 on both tests formed an age-typical WM group. The ages of the children in the two groups were matched to within 30 days of 50 children in the ADHD group (mean ages: ADHD, *M* = 9 years, 9 months, *SD* = 12.64, comparison, *M* = 9 years 10 months, *SD* = 11.98, low WM, *M* = 9 years, 9 months, *SD* = 12.11). None of the children had a diagnosis of ADHD. Ethical approval was obtained through both the local National Health Service and Durham University's ethics boards. Consent was obtained from parents/guardians and children, with appropriate opportunities for withdrawal.

### Procedure

Testing took place on between two and five individual testing sessions according to the individual child, with a total testing time of approximately 4 h. Regular breaks were introduced as required to reduce fatigue and optimize compliance. All assessments were conducted in a quiet area of the child's school. Children with ADHD receiving drug treatment ceased ingestion at least 24 h prior to testing. As all prescribed drugs were fast release, their physiological effects were eliminated at the time of test. Both children and their teachers were asked to verify that no medication had been taken prior to testing. In line with the administration guidelines of the executive function test battery, some children in the ADHD and low WM groups did not complete the tests either due to poor performance on practice trials and/or tasks measuring the baseline component processes necessary for a higher-level task (between two and eight out of 83 in the ADHD group and between one and six in the low WM group, depending on the test). All children in the comparison group completed every task. Teacher behavior ratings assessing ADHD symptoms were returned for 71%, 54% and 46% of the ADHD, low WM and comparison groups, respectively. Ratings of executive function problem behaviors were returned for 55% of the ADHD sample, 50% of the low WM group and 38% of the comparison group.

### Measures

#### Working memory

The AWMA (Alloway, 2007) provided multiple tests of verbal STM (Digit Recall, Word Recall, Non-word Recall), visuo-spatial STM (Dot Matrix, Block Recall, Mazes Memory), verbal WM (Backward Digit Recall, Listening Recall, Counting Recall), and visuo-spatial WM (Mr X, Spatial Span, Odd One Out). All tests yield standard scores.

#### Executive functions

***Switching***. The Number-Letter Sequencing test of the Delis-Kaplan Executive Function System (D-KEFS, Delis et al., 2001) assesses set-shifting/switching. The higher-level executive condition, Number-Letter Sequencing, requires children to connect letters and numbers in a progressive increasing alternating sequence (*A-1-B-2-C-3*, etc.). Other baseline conditions within this test, Visual Scanning, Motor Speed, Number Sequencing, and Letter Sequencing, measure the basic processes involved in Number-Letter Sequencing. Completion times are calculated for each condition and converted to scaled scores. Errors are represented as cumulative percentiles for baseline conditions and as a scaled score for the switching measure. A scaled contrast score represents differences in performance between the baseline and switching measure.

***Inhibition and inhibition with switching***. The Color Word Interference and Color Word Intereference with Switch tasks (D-KEFS, Delis et al., 2001) measure inhibitory control. The Color Word Interference condition involves a standard Stroop task in which the child inhibits the over-learned verbal response of naming a color word, and instead names the ink color. The Color Word with Switch condition involves two executive functions: inhibition and switching. The child is instructed to name the color of the ink for all words except those displayed in a box; on these trials, the task is to name the color word rather than the ink color. Further baseline conditions, Color Word Reading and Color Naming, assessed relevant processing abilities. Completion times are converted to scaled scores for all tasks. Errors are scored as cumulative percentiles for baseline tasks and scaled scores for the executive tasks.

***Sorting***. The D-KEFS Sorting Test (Delis et al., 2001) measures problem-solving and conceptual learning. It requires children to sort, or describe the sorting categories of, six cards according to different dimensions—color, shape or pattern on the cards, characteristics (upper or lower case, number of letters/syllables) or semantic information about words printed on the cards. In one condition, Free Sort, the child sorted the cards into as many categories as possible. Both the number of correct sorts and level of the description of each sort were scored. In a second condition, Recognition, the examiner sorted the cards into different categories and asked the child to describe what principles had been used to sort the cards. Both the number of correct sorts and the descriptions of the sorts were converted to scaled scores.

***Planning***. Planning and the ability to inhibit an impulse response were measured using the Tower Test (D-KEFS, Delis et al., 2001), in which the child moves 5 disks of different sizes arranged on three pegs from a start position to an end state one disk at a time without placing any disk on a smaller disk. Total achievement and time per move scores are converted to scaled scores. Total rule violations are scored as cumulative percentiles.

***Sustained attention***. The *K*-test of the Continuous Performance Test (CPT, Conners and Multi-Health Staff, 2004) assesses sustained attention. In this task, a series of 480 letters appear on the computer screen at a rate of 1 per second. The child's task is to press the space bar only when a *K* is displayed, which occurs on 140 of the trials at random intervals. The following measures are obtained: average response delay in ms, the numbers of omissions (possible range 0–140) and commissions (possible range 0–480), and total accuracy (proportion of trials correct).

***Response suppression***. Motor response inhibition was measured using the Walk/Don't Walk subtest from the Test of Everyday Attention for Children (TEA-Ch, Manly et al., 1999). The child is given a sheet showing paths made up of footprints and has to dot the next footprint on the path with a marker pen when they hear a frequently occurring “go” sound. The child is instructed not to dot the next footprint when an occasional “no go” sound is played, thereby inhibiting the prepotent go response. Correct responses are converted to a scaled scores.

***Reading and mathematics***. The basic reading, spelling and reading comprehension subtests of the Wechsler Objective Reading Dimensions (Wechsler, 1993), and both subtests of the Wechsler Objective Number Dimensions (Wechsler, 1996), mathematical reasoning and number operations, were administered. In each case, standard scores were calculated.

***IQ***. The four subtests of the Wechsler Abbreviated Scales of Intelligence (WASI, Wechsler, 1999) were administered: Block Design, Matrix Reasoning, Similarities and Vocabulary. Verbal and Performance IQ standard scores were calculated from subtest scores.

#### Behavior

***Teacher rating scale***. Classroom teachers completed the Conners Teacher Rating Scale Revised Short-Form (CTRS-R, Conners, 1997) for each child. Teachers rate 28 statements as not true at all (0), just a little true (1), pretty much true (2) or very much true (3). These statements comprise four subscales, which provide an index of oppositional behavior, cognitive problems and inattention, hyperactivity and ADHD symptoms. *T*-scores were calculated.

***Behavior rating inventory of executive function***. The Behavior Rating Inventory of Executive Function (BRIEF, Gioia et al., 2000), consisted of 86 statements, which teachers rated as occurring never (1), sometimes (2), or often (3). These statements formed eight subscales designed to assess executive functioning in the school environment as follows: Inhibit- the ability to control impulses; Shift—the ability to move freely from one situation, or aspect of a problem, to another; Emotional Control—the ability to modulate emotional responses; WM—the ability to hold in mind information for the completion of an activity; plan/organize—the ability to set goals, develop appropriate steps ahead of time and anticipate future events; Initiate—the ability to begin a task and work independently; Organization of Materials—the ability to maintain parts of the environment in an orderly manner; Monitor—the ability to assess performance, check work and keep track of effort. Three composite scores were derived: Behavioral Regulation Index, Metacognitive Index and Global Executive Score. *T*-scores were calculated for each scale and composite index.

## Results

Descriptive statistics for the cognitive measures are displayed by group in Table 1 and behavior ratings are shown in Table 2. Separate multivariate analyses of variance (MANOVA) were conducted for those tests that generated multiple dependent variables on a comparable scale: WM, IQ, reading, mathematics, and teacher behavior ratings. Univariate *F*-tests compared performance between groups on individual measures. Where there were significant group differences, pairwise comparisons were conducted for each of the three pairwise group combinations. Bonferroni corrections were applied to correct for multiple testing, yielding significance thresholds of *p* < 0.0125 for WM composite scores, sustained attention, and Conners behavior ratings, *p* < 0.004 for individual WM subtests, and BRIEF behavior scores, *p* < 0.005 for indices derived from the switching task, *p* < 0.005 for the inhibition and combined inhibition with switching task measures; *p* < 0.016 for sorting, planning and IQ, and *p* < 0.01 for reading and mathematics.

**Table 1:** **Cognitive measures as a function of group**.

	**ADHD**			**Comparison**			**Low WM**			**Group comparison**			**Comparison vs. ADHD**		**Comparison vs. Low WM**		**ADHD vs. Low WM**	
	***n***	***M***	***SD***	***n***	***M***	***SD***	***n***	***M***	***SD***	***F***	***P***	***partial eta***	***p***	***d***	***p***	***d***	***p***	***d***
**WORKING MEMORY**																		
Digit recall	83	94.735	15.538	50	104.420	12.977	50	89.360	13.363	14.383	0.000	0.138	0.000	0.670	0.000	1.144	0.044	0.372
Word recall	83	98.807	18.204	50	112.560	13.109	50	93.840	15.401	18.367	0.000	0.169	0.000	0.818	0.000	1.313	0.109	0.296
Nonword recall	83	103.084	16.545	50	113.220	12.334	50	96.480	16.902	14.653	0.000	0.14	0.000	0.606	0.000	1.145	0.029	0.395
Verbal STM	83	98.819	16.808	50	112.260	10.236	50	92.360	15.122	23.813	0.000	0.209	0.000	0.842	0.000	1.570	0.028	0.405
Dot matrix	83	90.699	17.873	50	105.360	18.154	50	84.720	19.091	17.200	0.000	0.160	0.000	0.793	0.000	1.108	0.071	0.323
Mazes memory	83	97.723	18.023	50	109.900	14.349	50	89.760	20.093	16.471	0.000	0.155	0.000	0.639	0.000	1.170	0.020	0.418
Block recall	83	87.988	18.679	50	111.060	16.138	50	86.960	22.069	27.634	0.000	0.235	0.000	1.132	0.000	1.262	0.775	0.050
Visuo-spatial STM	83	90.602	18.937	50	110.240	14.682	50	84.640	20.785	27.240	0.000	0.232	0.000	0.989	0.000	1.444	0.092	0.300
Listening recall	83	90.651	17.698	50	102.760	10.843	50	75.660	8.412	47.572	0.000	0.346	0.000	0.928	0.000	2.815	0.000	1.148
Counting recall	83	87.482	17.532	50	101.160	13.555	50	81.620	14.828	20.485	0.000	0.185	0.000	0.845	0.000	1.377	0.050	0.362
Backward digit recall	83	89.241	14.211	50	105.980	9.612	50	81.780	8.036	57.959	0.000	0.392	0.000	1.505	0.000	2.742	0.001	0.671
Verbal WM	83	86.759	17.031	50	104.340	8.905	50	75.260	10.472	58.723	0.000	0.395	0.000	1.278	0.000	3.002	0.000	0.836
Odd one out	83	88.253	17.139	50	105.720	15.863	50	81.500	13.411	31.771	0.000	0.261	0.000	1.144	0.000	1.655	0.019	0.442
Mr X	83	85.843	14.675	50	102.860	19.246	50	82.040	13.219	25.996	0.000	0.224	0.000	1.220	0.000	1.283	0.136	0.273
Spatial span	83	82.819	16.129	50	98.180	16.319	50	78.180	14.931	22.455	0.000	0.200	0.000	0.989	0.000	1.280	0.101	0.299
Visuo-spatial WM	83	82.928	15.533	50	102.840	18.109	50	78.120	12.501	36.950	0.000	0.291	0.000	1.421	0.000	1.615	0.066	0.343
**EXECUTIVE FUNCTION SWITCHING**																		
Visual scanning time	83	11.217	2.846	50	11.840	2.706	50	10.800	3.084	1.664	0.192	0.018						
Visual scanning omissions	83	69.169	40.783	50	78.600	34.811	50	68.680	41.091	1.088	0.339	0.012						
Visual scanning commissions	83	95.422	20.470	50	96.200	18.805	50	96.200	18.805	0.036	0.965	0.000						
Motor speed time	83	10.783	2.701	50	11.160	2.510	49	9.469	3.355	4.966	0.008	0.053						
Letter sequencing time	83	9.060	3.759	50	9.920	2.989	47	7.787	3.901	4.299	0.015	0.046						
Letter sequencing errors	83	60.554	45.490	50	85.760	33.007	47	64.319	45.895	5.797	0.004	0.061	0.001	0.552	0.009	0.543	0.652	0.082
Number sequencing time	83	10.000	3.298	50	10.580	2.942	49	8.000	3.857	8.229	0.000	0.084	0.309	0.162	0.000	0.759	0.002	0.559
Number Sequencing errors	83	85.036	34.940	50	96.160	19.003	49	90.102	29.672	2.175	0.117	0.024						
Number-letter sequencing time	80	10.513	2.851	50	10.560	2.922	44	9.500	3.114	2.025	0.135	0.023						
Number-letter sequencing errors	80	8.163	3.820	50	10.340	2.200	44	7.630	3.511	9.323	0.000	0.097	0.000	0.594	0.000	0.949	0.440	0.145
Number-letter sequencing contrast	80	10.400	2.809	50	9.900	2.485	44	11.205	3.188	2.532	0.082	0.029						
**INHIBITION AND INHIBITION SWITCHING**																		
Color naming time	83	10.133	2.991	50	12.180	3.415	49	8.878	3.557	12.993	0.000	0.127	0.000	0.625	0.000	0.947	0.032	0.383
Color naming errors	83	44.795	37.309	50	72.800	35.487	49	58.429	41.910	8.538	0.000	0.087	0.000	0.707	0.068	0.371	0.055	0.344
Word reading time	83	9.735	3.433	50	12.140	2.129	46	9.543	2.722	12.811	0.000	0.127	0.000	0.782	0.000	1.071	0.745	0.062
Word reading errors	83	52.663	44.738	50	82.800	33.123	46	61.957	44.691	8.144	0.000	0.085	0.000	0.674	0.011	0.536	0.260	0.208
Color-word interference time	82	10.402	3.243	50	11.680	2.917	45	9.378	3.017	6.623	0.002	0.071	0.024	0.408	0.000	0.776	0.083	0.327
Color-word interference errors	82	6.671	5.840	50	10.740	2.926	45	7.756	4.096	11.570	0.000	0.117	0.000	0.819	0.000	0.850	0.271	0.218
Color-word interference contrast	80	9.950	3.190	50	9.340	2.047	45	9.933	3.414	0.737	0.480	0.008						
Color-word interference with switch time	77	10.545	3.247	49	12.286	2.865	44	9.886	2.871	8.023	0.000	0.088	0.003	0.569	0.000	0.837	0.265	0.215
Color-word interference with switch errors	77	6.766	4.510	49	10.429	2.958	44	7.818	4.150	12.531	0.000	0.130	0.000	0.846	0.001	0.734	0.207	0.243
Color-word interference with switch contrast	77	10.039	3.266	49	9.755	1.797	44	10.136	3.167	0.228	0.796	0.003						
**PROBLEM-SOLVING**																		
Free sorts	83	5.711	2.487	50	7.860	2.259	50	5.060	2.535	18.594	0.000	0.171	0.000	0.856	0.000	1.168	0.149	0.259
Free sorts desciption	83	6.494	2.661	50	8.660	2.264	50	5.700	2.750	17.992	0.000	0.167	0.000	0.801	0.000	1.181	0.102	0.293
Recognition sorting	83	7.024	2.992	50	9.660	2.738	50	7.880	11.826	2.460	0.088	0.027						
**PLANNING**																		
Time per move	83	13.253	3.787	50	11.840	1.777	50	12.220	2.013	4.206	0.016	0.045	0.015	−0.487	0.319	−0.201	0.077	0.356
Total achievement	83	13.952	4.796	50	12.940	3.830	50	12.540	4.879	1.685	0.188	0.018						
Rule violations	83	21.928	35.308	50	61.540	41.108	50	38.600	39.327	16.935	0.000	0.158	0.000	1.061	0.005	0.570	0.013	0.447
**SUSTAINED ATTENTION**																		
Average response time (ms)	83	423.072	95.281	50	446.588	64.718	50	445.744	80.951	1.714	0.183	0.019						
Omissions (counts)	83	34.386	23.071	50	23.360	21.211	50	40.560	29.397	6.383	0.002	0.066	0.007	−0.420	0.001	−0.680	0.181	0.235
Commissions (counts)	83	110.578	82.703	50	48.040	51.650	50	74.400	74.359	11.956	0.000	0.117	0.000	−0.796	0.042	−0.418	0.012	0.461
Accuracy (%)	83	0.749	0.170	50	0.834	0.147	50	0.715	0.200	6.412	0.002	0.067	0.004	0.458	0.001	0.688	0.294	0.186
**RESPONSE SUPPRESSION**																		
Accuracy	83	3.831	3.312	50	9.280	3.687	50	4.140	3.574	42.555	0.000	0.321	0.000	1.582	0.000	1.416	0.614	0.090
**IQ**																		
Verbal IQ	83	89.458	13.378	50	102.200	14.077	50	86.420	14.750	18.728	0.000	0.172	0.000	0.906	0.000	1.095	0.225	0.216
Performance IQ	83	91.602	14.056	50	99.860	11.477	50	85.200	12.171	16.282	0.000	0.153	0.001	0.630	0.000	1.240	0.008	0.488
Full scale IQ	83	89.843	13.304	50	101.080	11.398	50	84.340	13.301	22.474	0.000	0.200	0.000	0.845	0.000	1.356	0.022	0.414
**READING AND MATHEMATICS**																		
Mathematical reasoning	83	87.783	14.381	50	101.380	10.725	50	85.320	14.937	21.083	0.000	0.190	0.000	0.928	0.000	1.252	0.347	0.168
Number operations	83	83.928	13.839	50	95.620	8.571	50	83.780	14.561	15.203	0.000	0.145	0.000	0.823	0.000	1.024	0.953	0.010
Basic reading	83	87.024	16.402	50	97.120	11.412	50	82.100	14.573	13.769	0.000	0.133	0.000	0.652	0.000	1.156	0.083	0.318
Spelling	83	86.747	14.411	50	100.120	12.411	50	85.140	15.254	17.960	0.000	0.166	0.000	0.902	0.000	1.083	0.543	0.108
Reading comprehension	83	82.530	12.534	50	97.100	10.359	50	80.080	13.608	29.457	0.000	0.247	0.000	1.115	0.000	1.420	0.292	0.187

**Table 2:** **Teacher behavior ratings, by group**.

	**ADHD**			**Comparison**			**Low WM**			**Group comparison**			**Comparison vs. ADHD**		**Comparison vs. Low WM**		**ADHD vs. Low WM**	
	***n***	***M***	***SD***	***n***	***M***	***SD***	***n***	***M***	***SD***	***F***	***P***	***partial eta***	***p***	***d***	***p***	***d***	***p***	***d***
**CONNERS**																		
Oppositional	59	65.085	15.355	23	50.957	11.109	27	55.630	13.021	9.979	0.000	0.158	0.000	0.996	0.183	0.387	0.007	0.666
Cognitive problems/inattention	59	60.797	11.522	23	46.652	6.005	27	64.333	11.228	20.058	0.000	0.275	0.000	1.243	0.000	2.052	0.187	0.311
Hyperactivity	59	62.237	11.920	23	46.696	4.704	27	53.593	11.982	18.589	0.000	0.260	0.000	1.300	0.013	0.827	0.003	0.723
ADHD index	59	63.136	13.923	23	47.826	7.203	27	58.037	13.221	12.184	0.000	0.187	0.000	1.128	0.002	1.000	0.113	0.376
**BEHAVIOR RATING INVENTORY OF EXECUTIVE FUNCTION**																		
Inhibit	46	70.261	16.046	19	46.579	6.239	25	58.880	18.072	18.169	0.000	0.283	0.000	1.388	0.009	1.012	0.006	0.667
Shift	46	65.435	15.947	19	49.263	6.332	25	55.440	13.629	11.812	0.000	0.206	0.000	1.094	0.059	0.619	0.008	0.676
Emotional control	46	70.478	19.718	19	50.158	9.347	25	59.000	19.740	10.693	0.000	0.189	0.000	1.030	0.060	0.608	0.019	0.582
Behavioral regulation index	46	70.913	17.247	19	48.105	7.310	25	58.560	18.157	15.917	0.000	0.261	0.000	1.288	0.023	0.821	0.005	0.698
Initiate	46	65.283	11.893	19	46.632	7.747	25	61.160	15.989	14.404	0.000	0.238	0.000	1.338	0.001	1.224	0.321	0.296
Working memory	46	68.000	15.338	19	48.474	7.396	25	63.520	17.154	13.566	0.000	0.226	0.000	1.202	0.001	1.226	0.273	0.276
Plan/Organize	46	66.500	12.466	19	47.105	7.971	25	57.920	12.913	17.767	0.000	0.279	0.000	1.528	0.005	1.036	0.010	0.676
Organization of materials	46	61.587	13.313	19	47.789	6.680	25	56.640	16.850	7.336	0.001	0.142	0.000	0.915	0.030	0.752	0.202	0.328
Monitor	46	69.826	12.129	19	47.000	8.800	25	62.880	19.417	18.550	0.000	0.287	0.000	1.447	0.002	1.126	0.056	0.440
Metacognition index	46	67.761	13.606	19	47.211	8.025	25	60.920	16.178	15.245	0.000	0.255	0.000	1.380	0.002	1.133	0.078	0.459
Global executive Score	46	69.935	15.187	19	49.684	13.221	25	62.040	18.902	11.308	0.000	0.204	0.000	1.188	0.018	0.769	0.059	0.463

### Cognitive measures

#### Working memory

A MANOVA revealed a significant group effect for composite WM scores, Hotelling's *T*^2^_(8, 352)_ = 16.567, *p* < 0.001. Univariate *F*-tests revealed significant group effects for all of the individual STM and WM subtests and the four derived composite scores. The ADHD and low WM groups performed significantly more poorly than the comparison group on all four WM component scores, and all 12 WM subtests. Performance was significantly lower for the low WM group than the ADHD group on two of three verbal WM tasks, Listening Recall and Backward Digit Recall, and for the verbal WM composite score. This is likely to reflect a selection artifact for the low WM group, as they were identified on the basis of low scores (<86) on these measures, and the two groups did not differ on the verbal WM task that was not used at screening, Counting Recall, when a Bonferroni correction was applied. No other significant differences were found between the low WM and ADHD groups.

#### Executive functions

***Switching***. Univariate analyses established significant group differences in two of the baseline tasks; Number Sequencing completion times and Letter Sequencing errors. There were no significant group differences in the other baseline conditions, Motor Speed and Visual Scanning. There were significant group differences in the frequency of errors on the higher-level switching task, Number-Letter Sequencing. However, the group difference in contrast scores, which reflect differences in performance between the baseline and higher-level conditions, was not significant.

Pairwise comparisons revealed that the low WM and ADHD groups made significantly more errors on the switching task than the comparison group. The ADHD group were also significantly less accurate than the comparison group in the baseline Letter Sequencing condition. Error rates did not differ significantly between the low WM and ADHD children on either the baseline or switching tasks. Group differences in completion time for the baseline Number Sequencing task were driven by significantly slower performance by the low WM group than either the ADHD or comparison groups.

***Inhibition control and inhibition with switching***. Significant group differences were established for all subtests of the Color-Word Interference and Color-Word Interference with Switch tasks, including the baseline and higher-level inhibition and inhibition with switch tasks. There were no significant group differences in contrast scores.

Subsequent pairwise comparisons revealed the ADHD group were both significantly slower and more errorful than the comparison group in the baseline Color Naming and Word Reading conditions and also on the higher-level inhibition with switch task, Color Word Interference with Switch. They also committed significantly more errors on the inhibition task than the comparison group, but completion times were not significantly different. Low WM children performed the baseline Color Naming and Word Reading tasks and the inhibition and inhibition with switch tasks significantly more slowly than the comparison group. They also made a significantly higher number of errors on both the higher-level executive tasks. There were no significant group differences between the ADHD and low WM groups on either of the baseline or higher-level subtests of Color Word Interference or Color Word Interference with Switch tasks.

***Sorting***. Significant group differences were observed for both the number and description of Free Sorts on the Sorting task, which measures problem-solving and conceptual learning skills. There were no significant group differences in the Recognition Sort condition. Where there were significant group differences, the comparison group performed at a significantly higher level than both the ADHD and low WM groups. The ADHD and low WM groups did not differ significantly on any measure.

***Planning***. There were no significant group differences in total achievement scores on the Tower Test. However, the ADHD group violated task rules significantly more often than both other groups, and also performed the task significantly faster than the comparison group. Time taken to complete the task between children with ADHD and those with low WM did not differ significantly. The low WM group made significantly more rule violations than children in the comparison group.

***Sustained attention***. Average response times for each trial did not differ between groups. There was, however, a significant group effect for accuracy that was accompanied by significant group differences for both error types. Overall, the ADHD and low WM children were significantly less accurate than the comparison group and both made a significantly greater number of omissions. The ADHD group additionally made significantly more commission errors than either of the other groups.

***Response suppression***. Significant group differences on the Walk/Don't Walk task reflected significantly poorer performance by both the ADHD and low WM groups relative to the comparison group. No significant differences were observed between those with ADHD and those with low WM.

***Reading and mathematics***. There was an overall significant group effect for academic ability, Hotelling's *T*^2(8, 352)^ = 7.227, *p* < 0.001. Performance was significantly higher for the comparison group than the other two groups across measures of mathematical reasoning, written number calculations, spelling, single word reading and reading comprehension. Reading and mathematics scores did not differ significantly between the low WM and ADHD groups.

***IQ***. A MANOVA revealed a significant group effect for IQ, Hotelling's *T*^2^_(6, 354)_ = 8.315, *p* < 0.001. Subsequent univariate *F*-tests established this difference was significant for both Verbal and Performance IQ, and for the derived Full Scale IQ score. Performance IQ scores were significantly higher for the comparison group than both other groups, and for the ADHD than the low WM group.

As non-verbal reasoning is highly associated with processing speed (Fry and Hale, 1996), a series of ANCOVAs were conducted to establish whether group differences between the low WM sample and the other groups were mediated by variation in Performance IQ scores. Consider first the differences between the low WM and comparison groups. Group differences were abolished in planning rule violations (*p* = 0.161), both the frequency of omissions (*p* = 0.255) and overall accuracy levels in sustained attention (*p* = 0.169), two of the visuo-spatial STM tasks, Dot Matrix (*p* = 0.006) and Mazes Memory (*p* = 0.005), time taken to complete the baseline Number Sequencing test of the switching task (*p* = 0.006) and both the higher-level inhibition (*p* = 0.017) and inhibition switching tasks (*p* = 0.015).

Group differences between the ADHD and low WM groups were largely unaffected by controlling for Performance IQ. The exception was Backward Digit Recall, on which the group difference was no longer significant (*p* = 0.009).

### Behavior ratings

#### Teacher rating scale

There was a significant effect of group on all four subscales of Conners behavior ratings, Hotelling's *T*^2^_(8, 204)_ = 8.552, *p* < 0.001. Teacher ratings of oppositional, inattentive and hyperactive behaviors were significantly elevated in the ADHD group relative to the comparison group. Both the ADHD and low WM groups received significantly elevated scores on the cognitive problems/inattention subscale and composite ADHD index. The ADHD group were rated as significantly more oppositional and hyperactive than the low WM group.

#### Behavior rating of executive function

Teacher ratings of behavioral difficulties related to executive function differed significantly by group, Hotelling's *T*^2^_(22, 152)_ = 2.978, *p* < 0.001. Significantly elevated symptoms were reported for each individual subscale of the BRIEF for the ADHD group relative to the comparison group. The low WM group received significantly higher ratings of problem behaviors on the Initiate, WM and Monitor subscales and on the composite Metacognition Index relative to the comparison group. There were no significant differences in teacher ratings between the ADHD and low WM children on any of the individual subscales or on the composite index scores of the BRIEF.

### Factor analysis

To investigate further the differences between the ADHD and low WM groups, a principal components analysis was conducted on the WM and executive function measures for all children (*N* = 183). Varimax rotation was used to force differentiation between factors and amplify group differences. To satisfy the recommended 10:1 case to variable ratio (Nunnally, 1978), a reduced set of variables was entered into the analysis. The measures were selected to provide speed and accuracy scores for each of the higher-level tasks: time and error scores for switching, inhibition, and inhibition switching; number of sorts for problem-solving; time per move and frequency of rule violations for planning; total score for response suppression; frequency of each of omission and commission errors and average response time per trial for sustained attention; a mean WM score derived from the four composite scores. This single score was selected in order to avoid entering multiple highly-correlated measures.

Three factors emerged with eigenvalues in excess of 1.00, explaining 29.396, 12.855 and 10.268% of variance, respectively. Factor loadings greater than 0.30 on the rotated factor matrix are shown in bold in Table 3. A broad range of executive measures loaded highly on Factor 1: WM, switching errors, inhibition and inhibition switching, problem-solving, both planning measures, response suppression, and omission in sustained attention. The measures loading most highly on Factor 2 were response times on the switching, inhibition and inhibition switching tasks, and to a lesser extent WM. This second factor is therefore predominantly associated with speed of processing. Factor 3 is linked with impulsivity in sustained attention, with high loadings of both the frequency of commission errors and response speed on the sustained attention task. A convergent three-factor solution was derived when oblique rotation was used.

**Table 3:** **Principal components analysis**.

		**Factor**		
		**1**	**2**	**3**
WM	Composite score	**0.757**	**0.328**	0.031
Inhibition	Time	0.109	**0.85**	0.123
	Errors	**0.704**	0.14	0.081
Inhibition switching	Time	0.136	**0.824**	0.044
	Errors	**0.669**	0.181	0.113
Switching	Time	0.086	**0.532**	−0.134
	Errors	**0.69**	−0.085	−0.019
Planning	Time	**−0.408**	0.223	0.063
	Errors	**0.558**	0.116	−0.253
Sustained attention	Omissions	**−0.451**	−0.243	0.225
	Commissions	−0.265	−0.074	**−0.879**
	Time	−0.004	−0.036	**0.912**
Response inhibition	Total score	**0.545**	0.214	0.048
Problem-solving	Total score	**0.661**	0.223	0.063

*Factor loadings greater than 0.3 are shown in bold. Solution derived using varimax rotation with Kaiser normalization. A convergent 3 factor solution is derived using oblique rotation*.

Factor scores are shown by group in Table 4. Univariate ANOVAs revealed significant group differences on all three factors: Factor 1, *F*_(2, 165)_ = 32.413, *p* < 0.01; Factor 2, *F*_(2, 165)_ = 7.548, *p* = 0.01; Factor 3, *F*_(2, 165)_ = 8.492, *p* < 0.01. *Post-hoc* exploration of the group differences established scores were significantly higher for the comparison group than both the ADHD and low WM groups on Factors 1 and 2. The low WM and ADHD groups did not differ on Factor 1 (executive functions). However, the low WM group had significantly lower scores than the ADHD group on Factor 2 (speed of processing). In contrast, the ADHD group scored more poorly than the low WM group on Factor 3 (impulsivity in sustained attention).

**Table 4:** **Factor scores displayed by group**.

	**ADHD**		**Comparison**		**Low WM**		**Comparison vs. ADHD**		**Comparison vs. Low WM**		**ADHD vs. Low WM**	
	***M***	***SD***	***M***	***SD***	***M***	***SD***	***p***	***d***	***p***	***d***	***P***	***d***
Factor 1	−0.364	0.976	0.824	0.575	−0.295	0.879	0.000	1.532	0.000	1.539	0.703	0.075
Factor 2	−0.013	0.923	0.374	0.989	−0.412	1.004	0.027	0.405	0.000	0.789	0.031	0.414
Factor 3	−0.318	1.163	0.164	0.691	0.392	0.791	0.010	0.520	0.145	0.308	0.001	0.727

Due to the disproportionately high number of boys in the ADHD group compared to both the low WM and comparison groups, a series of 3 × 2 ANOVAs with group (ADHD, low WM and average WM) and gender (boys and girls) were conducted to test for gender effects. There were no significant group x gender interactions for any of the cognitive measures, teacher rating scores or factor scores (all *p*s > 0.05).

## Discussion

This study provides the first comprehensive comparison of WM, executive function, academic ability and classroom behavior in children with low WM and those with ADHD. Both groups were characterized by overlapping patterns of WM and executive function deficits compared with both a typically developing comparison group drawn from the same population as the low WM children and population-based test standardizations. There were two important differences between the groups. First, the low WM children were slower to respond on several tasks. Second, the ADHD group were more hyperactive and exhibited more difficulties in controlling impulsivity in sustained attention.

First we consider the common characteristics of the two groups. They were judged as having equivalently high levels of inattentive behavior. For the ADHD group this is expected, as inattention is one of the defining characteristics of the combined subtype. It also replicates previous reports of inattentive behavior in low WM groups (Kane et al., 2007; Gathercole et al., 2008a; McVay and Kane, 2009), although the parity in the degree of rated inattentiveness across the two groups is novel and worthy of note. Their WM characteristics were also highly similar, in terms of both the profile and magnitude of impairments. Compared both with the typically-developing group and age norms, children with low WM and those with ADHD had substantial deficits in tests of visuo-spatial STM, verbal WM and visuo-spatial WM. Their verbal STM scores fell within the age-typical range. The low WM group scored more poorly than the ADHD group on the two verbal WM tasks used to identify them at screening. However, they did not differ on a third measure that was administered after screening. This suggests that the group differences on individual measures reflected sampling artifacts. Although the low WM group were selected on the basis of poor performance on two verbal tests of WM, there was no evidence for domain-specific impairments. Their composite verbal WM scores (75.26) were equivalent to their visuo-spatial WM scores (78.12) and their IQ profile was flat across both verbal and non-verbal assessments (standard scores 86.42 and 85.20 respectively).

The general pattern of findings is consistent with a common impairment in both groups in the domain-general executive control aspect of WM (Bayliss et al., 2003; Kane et al., 2004; Alloway et al., 2006), but not in verbal STM. The low performance on visuo-spatial STM tests could reflect a particular deficit in the visuo-spatial sketchpad. Alternatively, on the basis of close links already reported between these measures and the central executive aspect of Baddeley's (2000) WM model (Miyake et al., 2001; Alloway et al., 2006; Burin et al., 2007), a more parsimonious interpretation could be a single underlying impairment in the central executive for both children with low WM and those with ADHD. Similar claims of central executive problems have already been made for both groups (e.g., Martinussen and Tannock, 2006; Gathercole et al., 2008a; Kofler et al., 2010; Kasper et al., 2012). The new finding here is the high correspondence between both the profile and severity of the WM impairments in this first direct comparison of the two samples.

Understanding the correspondences between deficits in the executive control aspect of WM and elevated ratings of inattentive behavior in these two groups raises the possibility that WM problems may be the cause of overt everyday problems in attentional focus. Consistent with Engle's model of WM (Kane et al., 2001), previous research supports a close relationship between controlled cognitive attention and WM. For example, adults with low WM spans are known to be poor at resisting distracting information in experimental tasks (Rosen and Engle, 1997; Kane and Engle, 2000; Conway et al., 2001). The current data extend these findings to suggest a link between poor WM and overt inattentive and distractible behaviors. This association has been previously observed in adults with low WM in cognitively-demanding everyday activities (Kane et al., 2007) and also in a community sample of unselected children where WM performance was correlated with levels of inattentive behavior (Lui and Tannock, 2007). The novel finding here is that these associations are present at the group level in children with poor WM and in children with ADHD. Low WM capacity may give rise to short attention spans and distractible behavior due to a failure to maintain in WM task goals, and also the intermediate products of the ongoing mental activity in order to achieve the goal, which causes attentional focus to shift away from the task in hand, either to other salient events in the environment or to internally-generated thoughts.

Impairments in the low WM and ADHD groups that were comparable in magnitude extended to other executive functions, too. High rates of problem behaviors across a wide range of executive function behaviors were reported by teachers for both the low WM and ADHD groups. They also performed poorly on direct measures of switching, inhibition, sorting, planning, sustained attention, and response suppression. These data extend previous findings in which problem behaviors relating to executive function have been reported in children with low WM (Gathercole et al., 2008a) and those with ADHD (e.g., Toplack et al., 2009). In a smaller sample selected similarly on the basis of low WM, St Clair-Thompson (2011) reported difficulties in direct measures of visual attention and planning, although not in switching, inhibition and response suppression in low WM children. With substantially greater statistical power, the present study establishes low WM children have a constellation of difficulties that extend considerably beyond WM to all assessed aspects of executive function.

The current findings are entirely consistent with patterns of pervasive executive function impairments and teacher reports of executive function problems in ADHD (e.g., Willcutt et al., 2005a; Toplack et al., 2009; Schoemaker et al., 2014). According to some theories of ADHD, executive function impairments underlie behavioral disturbances and are central to the disorder (Barkley, 1997; Zelazo and Muller, 2002). Consistent with this, results from a meta-analysis of structural neuroimaging studies indicate that ADHD is associated with neuroanatomical abnormalities in areas of the brain related to executive function (Valera et al., 2007).

There are a number of possible reasons for why the common cognitive deficits of the groups might be so pervasive. First, difficulties may be present in all executive functions, including WM, because they rely on the same frontal brain networks (Duncan and Owen, 2000). Any impairment to this network would be expected to disrupt multiple executive functions. An alternative possibility is that WM deficits may play a causal role in other executive tasks and related behaviors. For example, low performance levels in both the low WM and ADHD groups on measures of inhibition or set switching may have resulted from the loss of crucial task relevant information or goals from WM (e.g., Kane et al., 2007; McVay and Kane, 2009). Finally, and in line with behavioral inhibition theories of ADHD, difficulties in inhibitory control may adversely affect WM and other executive functions due to problems inhibiting task irrelevant information and regulating goal-directed behavior (Barkley, 1997).

However, a degree of caution is necessary in interpreting these findings simply in terms of widespread problems in the broad executive control system. This is because in some cases, children with ADHD and low WM had deficits on component (non-executive) measures that were as great as those observed on measures requiring higher-order executive control. For example, both groups had problems in simple sequencing, word reading and color naming. Thus, difficulties in the inhibition and switching conditions requiring executive control may have been mediated by problems in basic processing, consistent with previous findings that when differences in basic processing skills are controlled, apparent executive impairments disappear both in ADHD and in children with reading difficulties (Rhodes et al., 2005; van Mourik et al., 2005; Willcutt et al., 2005b; Marzocchi et al., 2008). The core deficits may therefore lie in more basic cognitive processes.

This study directly compared the academic achievements of children with low WM and those with ADHD. Poor scholastic performance has been previously reported for each group separately (Loe and Feldman, 2007; Gathercole and Alloway, 2008). These findings were replicated, and it was additionally found that underachievement was equivalent in the two groups even when group differences in IQ were taken into account. This has important educational implications as enhanced learning support was provided in school to all children in the ADHD sample but not to the low WM group. Warner-Rogers and colleagues suggest that children who find it hard to pay attention, but who are not disruptive in the classroom are at risk for being neglected in educational settings (Warner-Rogers et al., 2000). The current observations of the low WM group underscore this point and demonstrate the need to identify and support learning in children with poor WM.

Critically, though, the low WM and ADHD groups were not indistinguishable in all respects. There were two key differences. First, children with low WM performed more slowly on several processing and executive functions tasks than both the comparison group and those with ADHD. This evidence for processing speed impairments in low WM is a novel and unexpected finding. It does not appear to be part of a broader problem in fluid intelligence (often associated with processing speed, Salthouse, 1996; Jensen, 1998), as controlling statistically for performance IQ had little impact on observed group differences. These difficulties may correspond to sluggish cognitive tempo (SCT), a set of symptoms strongly associated with the predominantly inattentive form of ADHD that includes high levels of daydreaming, slow response times, poor mental alertness and hypoactivity (e.g., Barkley, 1990; McBurnett et al., 2001). Although not included in the DSM criteria for ADHD, SCT symptoms have been advanced as a marker for a subgroup of individuals with the inattentive form of ADHD who have a distinct primarily inattentive disorder (Carlson and Mann, 2002; Hinshaw et al., 2002; Hartman et al., 2004; Huang-Pollock et al., 2005). It is therefore possible that children with low WM may correspond to those with the predominantly inattentive subtype of ADHD, a diagnosis that is not commonly applied in child psychiatric services in the UK.

Secondly, ADHD children made more errors than the low WM group on some of the tasks that required the inhibition of impulsive motor responses. In particular, they violated rules more frequently during a planning task, and made more commission errors (responding impulsively to non-target stimuli) on the Continuous Performance Test of sustained attention. This is one of the most widely reported deficits in ADHD (e.g., Epstein et al., 2003; Willcutt et al., 2005a; Rubia et al., 2007, 2009), and the present data indicates that it has a high degree of specificity to ADHD. Elevated levels of impulsivity in ADHD children were also reflected in the teacher ratings of high levels of hyperactive, impulsive and oppositional behaviors. Although there were more boys in the ADHD sample these group differences, and those in the cognitive assessments, were not mediated by gender. Gender ratios in ADHD are dependent on whether the sample is drawn from clinical (referred by psychiatrists) or community-based settings (APA, 2002), with a more equal balance of boys and girls common among samples selected via routine population-based screening (Gaub and Carlson, 1997; Arica and Conners, 1998). In the current study, the ADHD sample was clinically referred whereas the low WM group were selected via routine classroom screening. These different routes to selection may explain why there were a disproportionately high number of boys in the ADHD group.

More generally, the observed pattern of shared general executive function disturbance combined with group-specific impairments in impulsive behavior in sustained attention (the ADHD group were more impulsive) and speed (the low WM children were slower) fits well with the proposal that ADHD arises from parallel disruptions in distinct cool (cognitively-based) and hot (affective, delay aversive) neurodevelopmental systems (Castellanos et al., 2006). The cool impairments in cognitive aspects of executive function (or possibly, more basic cognitive processes) and WM that are linked also with inattention (Castellanos et al., 2006) are present both in the children with ADHD and low WM, whilst the hot difficulties in controlling aversion to delay in attention-demanding tasks appears to be restricted to individuals with ADHD. The additional difficulty in slow response times observed only in the low WM group does not fit within this ADHD framework, but may be symptomatic of a subgroup of children with the predominantly inattentive form of ADHD who are characterized by SCT (Hartman et al., 2004).

In summary, this study is the first to demonstrate that children with low WM and those with ADHD have largely equivalent problems across a wide range of measures of basic and higher-level cognitive functioning, and in particular in behaviors associated with executive functions. The groups are also indistinguishable in terms of their poor learning progress in mathematics and reading. There were important differences, too. The low WM children were slower to respond than the ADHD group across tests, and the ADHD children were more hyperactive and impulsive in their behavior and in some aspects of controlling responses when required to sustain attention. These distinctions may have considerable practical value for practitioners working with developmental populations. First, despite the striking differences in classroom management challenges raised by children with ADHD (high) and those with low WM (low), their needs for educational support as indexed by low levels of attainment are equivalent and may warrant similar levels of resourcing (e.g., Warner-Rogers et al., 2000). Second, the two groups may also respond similarly to interventions that address their shared problems in WM and executive functions, as we have found with WM training (Holmes et al., 2009, 2010; Dunning et al., 2013). It may also be expected that new methods such as strategy training already shown to enhance memory function in children with low WM (St Clair-Thompson et al., 2010) will be similarly beneficial in ADHD. However, the special behavioral difficulties that distinguish children with ADHD are likely to require different kinds of intervention that target psychosocial rather than cognitive skills, such as behavioral modification (e.g., Fabiano et al., 2009). For children with ADHD, a synergistic approach combining interventions targeted at both cognitive and behavioral problems might be optimal (e.g., Rapport et al., 2013). We suggest that adopting a multi-dimensional approach to profiling individual children with WM and related executive function problems—for example, by distinguishing executive from impulsive problems—may provide a valuable means of identifying those interventions, which either singly or in combination, are likely to be effective for individual children.

### Conflict of interest statement

The authors declare that the research was conducted in the absence of any commercial or financial relationships that could be construed as a potential conflict of interest.
